# Mitochondrial Metabolism in Major Neurological Diseases

**DOI:** 10.3390/cells7120229

**Published:** 2018-11-23

**Authors:** Zhengqiu Zhou, Grant L. Austin, Lyndsay E. A. Young, Lance A. Johnson, Ramon Sun

**Affiliations:** 1 Molecular & Cellular Biochemistry Department, University of Kentucky, Lexington, KY 40536, USA; zhengqiu.zhou@uky.edu (Z.Z.); grant.austin@uky.edu (G.L.A.); Lyndsay.young@uky.edu (L.E.A.Y.); 2 Department of Physiology, University of Kentucky, Lexington, KY 40536, USA; Johnson.Lance@uky.edu

**Keywords:** metabolism, mitochondria, Alzheimer’s disease, epilepsy, traumatic brain injury

## Abstract

Mitochondria are bilayer sub-cellular organelles that are an integral part of normal cellular physiology. They are responsible for producing the majority of a cell’s ATP, thus supplying energy for a variety of key cellular processes, especially in the brain. Although energy production is a key aspect of mitochondrial metabolism, its role extends far beyond energy production to cell signaling and epigenetic regulation–functions that contribute to cellular proliferation, differentiation, apoptosis, migration, and autophagy. Recent research on neurological disorders suggest a major metabolic component in disease pathophysiology, and mitochondria have been shown to be in the center of metabolic dysregulation and possibly disease manifestation. This review will discuss the basic functions of mitochondria and how alterations in mitochondrial activity lead to neurological disease progression.

## 1. General Mitochondrial Function

### 1.1. Mitochondria: Metabolic Hub of a Cell 

The mitochondrion is the result of evolution from a perfect marriage of an α-proteobacterium and a precursor of a modern eukaryotic cell, evidenced by it being the only non-nuclear sub-cellular organelle that has its own DNA [1]. In mammals, mitochondria are passed down maternally [2] and they are present in every cell in the body, except red blood cells [3]. Mitochondria are bilayer organelles with an outer and inner membrane that enclose the intermembrane space and a matrix compartment, respectively [4]. Mitochondrial DNA (mtDNA) are circular and reside within the matrix. Furthermore, mtDNA are intron-free which make them more susceptible to mutagenesis than nuclear DNA [5]. The mitochondrial proteome consists of over 3300 proteins and more are being identified daily [6]. mtDNA encode for 13 proteins that are part of the electron transport chain, which produces ATP via oxidative phosphorylation [7]. Transport of nuclear-encoded proteins to either the matrix or the intermembrane space requires separate signals. Matrix localization signals are located on the N-terminal of a protein and transport to the matrix requires membrane potential and ATP hydrolysis [8]. The intermembrane translocation signal is a hydrophilic region internal to the cell membrane that directs protein localization independent of membrane potential or ATP hydrolysis [9,10]. The intermembrane space is home to proteins important for mitochondrial structural integrity and multiple proteins in the BCL-2 family that control programmed cell death or apoptosis [11,12]. The intermembrane space and mitochondrial matrix contain proteins for the tricarboxylic acid (TCA) cycle—the major metabolic hub for cellular homeostasis and the electron transport chain that generates ATP from the redox gradient [13,14,15]. During ATP production, mitochondria generate a large number of reactive oxygen species (ROS) that are contained within the matrix [16]. Controlled release of ROS supports signaling events [17,18,19]; however, ectopic release of ROS from the mitochondria can result in DNA, RNA, and protein damage that ultimately leads to cell death [20,21]. Recent advances in techniques such as real-time oxygen consumption monitoring [22] and stable isotope enriched metabolomics have revealed an enormous amount of information on mitochondrial metabolism and its connection to cellular physiology [23,24]. These studies confirm that mitochondrion is an extremely complex and dynamic organelle and is at the crossroads of cellular metabolism and signaling. 

### 1.2. Mitochondria and Energy Production

**Figure 1:**
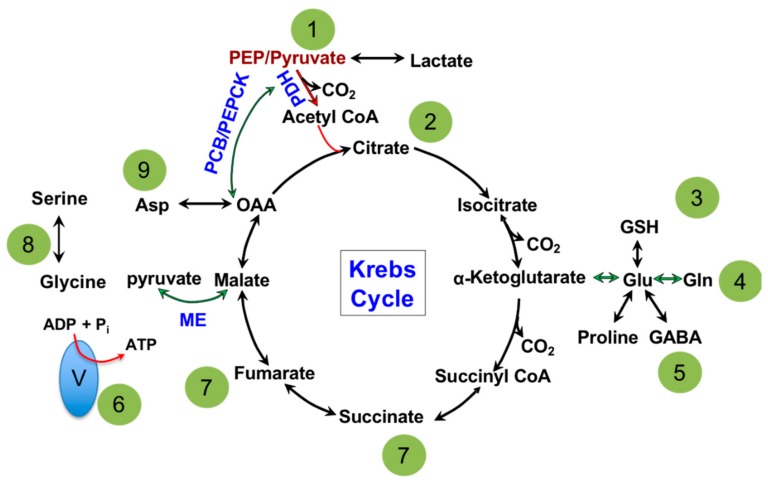
Major pathways and metabolite exchange that take place in the mitochondria. 1: Glycolysis and gluconeogenesis connects to the mitochondria by phosphoenolpyruvate (PEP), pyruvate and lactate. 2: Fatty acid biosynthesis and oxidation utilize citrate and acetyl-CoA as metabolic intermediates. 3: Glutathione (GSH) is produced in the mitochondria from glutamate, which maintains cellular redox balance. 4: Protein biosynthesis uses de novo synthesized amino acids from the mitochondria. 5 Neurotransmitter biosynthesis (proline and GABA) partially takes place in the mitochondria then continues in the cytosol. 6: Electron Transport Chain subunits I-V make up the Oxidative Phosphorylation pathway and are located on the inner mitochondrial membrane and use reducing equivalents, NADH and FADH_2_, as electron sources. Electrons move through the subunits from I or II to IV, which creates a proton gradient in the process. The proton gradient is used by subunit V to generate ATP. 7: Fumarate and succinate are exported to the cytosol as enzyme co-factors. 8: 1-carbon metabolism supplies carbon for purine nucleotide biosynthesis and methylation of proteins and DNA. 9: Aspartate (Asp) supports pyrimidine ring biosynthesis. →: Canonical oxidative pathway. →: Anaplerotic pathway. Pyruvate dehydrogenase (PDH); pyruvate carboxylase (PCB); phosphoenolpyruvate carboxykinase (PEPCK); malic enzyme (ME); gamma-aminobutyric acid (GABA).

The major biochemical pathway in the matrix of a mitochondrion is the TCA cycle, shown in Figure 1. Dr. Hans Adolf Krebs won the Nobel Prize in Physiology or Medicine in 1953 for its discovery; hence, it is also referred to as the Krebs cycle [25]. Dr. Krebs discovered that all of the enzymes in the TCA cycle are bi-directional in cell-free assays [25] which led to the illustration of the circular pathway seen in biochemical textbooks. The primary role of the TCA cycle is to extract electrons from carbon sources in the form of NADH and FADH_2_ and supply them to the electron transport chain (ETC) for the production of ATP [26,27]. Cytoplasmic metabolites supply the TCA cycle to facilitate its continuous operation and are divided into two categories: (1) the canonical oxidative pathway and (2) anaplerotic pathways. The canonical oxidation pathway is a continuation from glycolysis, where pyruvate enters the TCA cycle as acetyl-CoA, carried out by the enzyme pyruvate dehydrogenase (PDH), which is then converted to citrate [27,28]. This is viewed as the major pathway to supply oxidative phosphorylation or ATP production. The anaplerotic pathways involve metabolic enzymes such as pyruvate carboxylase (PCB) [29], phosphoenolpyruvate carboxykinase (PEPCK) [30], malic enzyme (ME) [31], glutaminase (GLS) [32] and glutamate dehydrogenase (GDH) [33]. While PCB, PEPCK and ME connect the TCA cycle with glycolysis; GLS/GDH supply carbon from glutamine. Anaplerotic pathways are bi-directional and most commonly believed to primarily maintain compartmentalized metabolite pools between the cytosol and mitochondria but not contribute to ATP production directly [34,35]. However, alternative studies have suggested that glutamine supports NADH production from the GLS/GDH anaplerotic pathway equally, if not more, when compared to the canonical pathway [36,37]. 

### 1.3. Mitochondria and Nucleotide Biosynthesis

**Figure 2:**
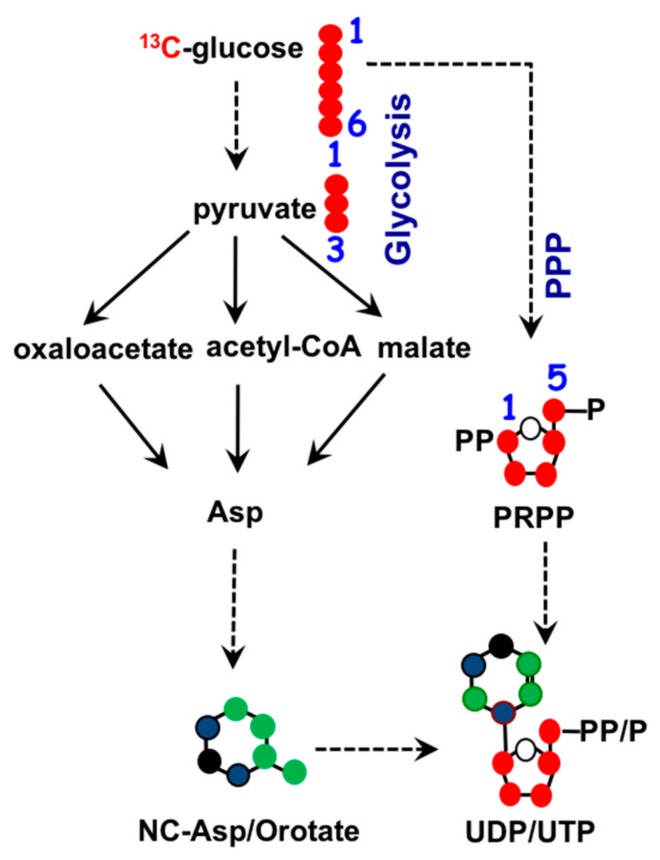
Biochemistry of pyrimidine nucleotide synthesis. Ribose base biosynthesis comes from the pentose phosphate pathway (PPP) in the cytosol. Mitochondrial TCA cycle metabolites oxaloacetate and aspartate (asp) supply three out of the four carbons in the diazine ring. •: Possible cytosolic-derived carbons, •: Possible mitochondrial-derived carbons. •: Nitrogen.

**Figure 3:**
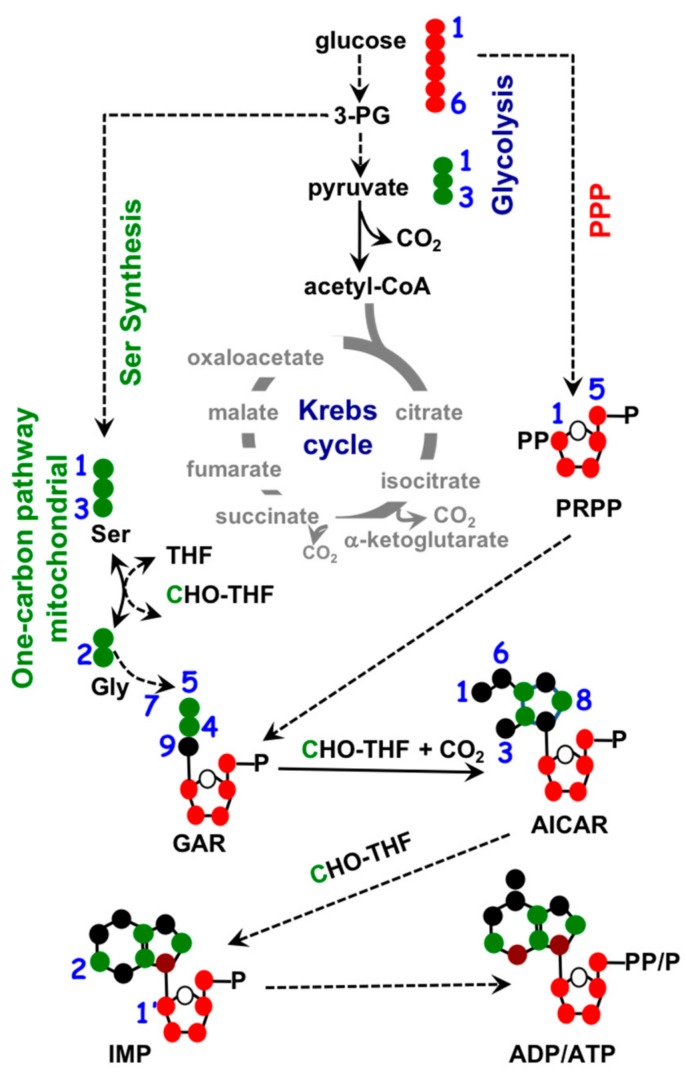
Biochemistry of purine nucleotide synthesis. Ribose base biosynthesis comes from pentose phosphate pathway (PPP) in the cytosol, mitochondrial 1-carbon metabolism contributes to purine ring biosynthesis through 5-methyltetrahydrofolate (CHO-THF). •: Possible cytosolic-derived carbons, •: Possible mitochondrial-derived carbons.

To maintain homeostasis during proliferation, a cell needs to produce energy, nucleotides, and fatty acids. All three biosynthetic pathways are connected to mitochondria through metabolite exchange. Energy production mainly stems from dehydrogenase activities within the TCA cycle, as mentioned earlier. Nucleotide biosynthesis also relies on key metabolites inside mitochondria. The two major classes of nucleotides are pyrimidines and purines. Pyrimidine nucleotides include cytosine, thymine, and uracil. Its structure consists of a ribose base connected to a 4-carbon diazine ring, see Figure 2. The ribose base is produced from the pentose phosphate pathway (PPP) that takes place in the cytosol. The biosynthesis of the diazine ring requires oxaloacetate, a TCA cycle intermediate, as a precursor. The first enzyme of this biochemical pathway is aspartate aminotransferase (GOT) [38] and its dominant isoform, GOT2, is primarily a mitochondrial enzyme [39]. Purine nucleotides include the key molecules adenine and guanine crucial for DNA and RNA biosynthesis as well as other biomolecules such as AMP, GMP, and NADH, as shown in Figure 3. Similar in structure to pyrimidines, purine nucleotides also contain a ribose base connected to a nitrogenous base produced from the PPP. Purine rings consist of a pyrimidine diazine fused to an imidazole ring. The 1-carbon or folate pathway donates carbon as 5-methyltetrahydrofolate (CHO-THF) to the biosynthesis of purine rings [40]. Key enzymes involved in this metabolic pathway include serine hydroxymethyltransferase (SMHT) [41], glycine decarboxylase (GDC) [42], and methylenetetrahydrofolate dehydrogenase (MTHFD) [43]. All three of these enzymes have mitochondrial isoforms expressed in a wide range of tissue types, suggesting the production of purine rings, at least in part, takes place in the mitochondria [44,45]. 

### 1.4. Mitochondria and Fatty Acid Metabolism. 

Fatty acids store energy, are precursors to lipid bilayers, and function as signaling molecules. De novo fatty acid synthesis and β-oxidation are opposing processes that either begin or end in the mitochondria. Acetyl-CoA and citrate are two key metabolites required for fatty acid synthesis. Citrate derived from glucose is transported out of the mitochondria and converted by ATP citrate lyase back to acetyl-CoA, the building block of fatty acids [46,47]. While fasting, stored fats undergo lipolysis to produce fatty acids for ATP production. During β-oxidation, free fatty acids are added to L-carnitine and transported into the mitochondria to be broken down sequentially to acetyl-CoA, which is then fed into the TCA cycle for ATP production [48,49]. Recent reports suggest that mitochondrial lipids such as lipoic acid, a co-factor for mitochondrial dehydrogenase, and 3-hydroxymyristyl-ACP, a structural component of complex I of the electron transport chain, are made de novo inside mitochondria in yeast [50]. To our knowledge, this process has yet to be studied in mammalian systems. 

### 1.5. Mitochondria Metabolism and Epigenetics 

Epigenetic programming is the way chromatin is organized. It has been shown to dictate cell fate [51], control the cell cycle [52], and contribute to disease pathology [53]. Post-translational modifications that alter gene expression patterns include histone acetylation, glycosylation, phosphorylation, and histone or DNA methylation. Histone acetylation occurs when the mitochondrial metabolite acetyl-CoA is added to the amino group of lysine residues on a histone. Acetyl-CoA is produced from pyruvate dehydrogenase, fatty acid oxidation, and exogenous acetate, processes that all take place inside mitochondria [54,55]. However, the exact relation between compartmentalized regulation of acetyl-CoA concentration to support both mitochondrial metabolism and histone acetylation remains unclear. Acetyl-CoA is also required for the production of uridine diphosphate N-acetyl-glucosamine (UDP-GlcNAc), a precursor for histone glycosylation [56]. The functional consequence of histone glycosylation is relatively unknown compared to other histone modifications but its level seems to correlate with extracellular glucose flux [57]. 

Levels of mitochondrial metabolic intermediates such as ATP and NAD^+^ can affect histone phosphorylation and acetylation, respectively. ATP is the substrate for histone phosphorylation by histone kinases [58] and NAD^+^ is a co-factor for the sirtuin family of deacetylases [59]. Mitochondria regulate NAD^+^ levels in two ways: first, through oxidative phosphorylation, where NAD^+^/NADH cycling takes place. Second, NAD^+^ is a purine nucleotide whose biosynthesis requires the serine-glycine 1-carbon metabolic pathway mentioned above. Furthermore, hydroxylase and demethylase activities are especially sensitive to changes in α-ketoglutarate (αKG), fumarate, and succinate concentrations. All three molecules are substrates crucial for the functioning of dioxygenases including prolyl hydroxylases and histone lysine demethylases, which are important for epigenetic regulation [60,61,62]. Germline or somatic mutations in mitochondrial enzymes that produce abnormal amounts of succinate [63], fumarate [64], and 2-hydroxyglutarate (2HG) [65], have been shown to have an inhibitory effect on these enzymes, leading to DNA hypermethylation-induced epigenetic alterations.

Mitochondrial metabolism controls the epigenetic landscape by providing substrates for acetylation, phosphorylation, and methylation of histones. The balanced supply of substrates from mitochondria to the nucleus is an extremely complex system that is tightly regulated by interconnected signaling pathways. Somatic mutations or altered mitochondrial metabolism by different diseased states greatly affect histone modification and the epigenetic landscape. Detailed mechanisms of how a cell can maintain the flow of metabolites between mitochondria and the nucleus to support proliferation, differentiation, and autophagy are relatively unknown and form an area of intense research.

## 2. Mitochondria Dysfunction in Alzheimer’s Disease

Alzheimer’s disease (AD) is a neurodegenerative disorder characterized by progressive and severe memory loss. AD is the most common form of dementia, affecting over 5.3 million people in the United States alone [66]. The strongest risk factor for AD is age, and as the elderly population expands, the number of individuals afflicted with this devastating disease is growing rapidly; particularly considering that effective therapies for the disease remain elusive [66]. The AD brain is characterized by two neuropathological hallmarks: extracellular senile plaques and intracellular neurofibrillary tangles [67]. The plaques are formed mostly from the deposition of amyloid β (Aβ) peptide, while neurofibrillary tangles are formed from neurofilaments and hyperphosphorylated tau protein. A small percentage of AD cases (<5%) are caused by mutations in genes encoding for either the amyloid β precursor protein (APP) or from mutations in genes encoding presenilin 1 or presenilin 2 (involved in the generation of amyloid beta (Aβ) from APP) [68]. These rare, autosomal dominantly inherited cases are typically referred to as familial or early-onset AD. However, the vast majority of AD cases occur sporadically in individuals >65 years of age and are commonly referred to as late-onset AD (LOAD) [67]. Despite the sporadic nature of LOAD, the inheritance of several well-established susceptibility genes significantly increases the risk of disease [69].

In addition to advanced age, various metabolic impairments predispose individuals to cognitive dysfunction and dementia, including LOAD. For example, metabolic dysfunction as in the case of insulin resistance and type 2 diabetes, increases the risk of dementia and shares several pathological characteristics with AD, such as neuroinflammation, increased oxidative stress, and cerebrovascular dysfunction [70,71]. Metabolic disorders also increase in incidence with age [72], meaning the number of high-risk elderly individuals suffering from metabolic disorders is expanding precipitously.

The central role of metabolism in AD is likely due to the fact that normal synaptic function requires a multitude of energy-intensive processes [73]. The first clues pointing toward a potential metabolic component of the disease were observed nearly four decades ago, as a series of neuroimaging studies utilizing flurodeoxyglucose positron emission tomography (FDG-PET) that showed that the brains of individuals with AD took up less glucose compared to those of cognitively normal controls [74,75,76,77]. Since these initial findings in the early 1980s, a multitude of additional studies have confirmed the phenomena, and a reduction in the cerebral metabolic rate of glucose (CMRglc), as measured by FDG-PET, is now considered one of the hallmarks of AD [78]. In fact, FDG-PET is able to differentiate AD from other types of dementia with a high degree of specificity due to specific regional signaling patterns [79]. Clinical AD symptoms essentially never occur without glucose hypometabolism, the extent of which strongly correlates with the severity of clinical symptoms [80,81,82]. Importantly, evidence suggests that these alterations in glucose metabolism occur very early in the neurodegenerative process [83,84,85,86], raising the possibility that metabolic dysfunction precedes, and possibly contributes causally, to the pathophysiology of AD. 

Despite these important findings, the molecular mechanisms underlying AD neurodegeneration remain elusive. The field has been dominated by the “amyloid hypothesis”, which argues that sequential cleavages of APP produce Aβ, initiating a cascade of neurodegeneration [87]. In this context, mitochondrial impairments observed in AD patients are thought to be secondary effects of Aβ toxicity, as Aβ may block mitochondrial translocation of ETC components [88,89,90], and impair respiratory chain function and oxidative phosphorylation in mitochondria [91,92]. Alternatively, some evidence points to a direct effect of mitochondria on AD neuropathology, which are either independent of Aβ or, themselves, potentially drive the changes in APP and Aβ homeostasis.

In support of the latter, shortly after the initial reports demonstrating the reduced glucose uptake via FDG-PET in AD patients, a series of additional PET-based studies described reductions in oxygen consumption in AD brains [93,94]. Importantly, around this same time, several groups began to show evidence of mitochondrial dysfunction in AD brain tissue. This included reductions in cytochrome oxidase (COX) activity in AD patients [88], and decreases in mitochondrial enzymes such as PDH, and other enzymes in the early part of the TCA cycle, such as isocitrate dehydrogenase and α-ketoglutarate dehydrogenase (αKGDH) [95,96]. Additionally, the activity of late TCA cycle enzymes such as succinate dehydrogenase and malate dehydrogenase were increased in AD brains, and the alterations of TCA cycle enzyme activities correlated strongly with clinical symptoms [97]. Together, these studies may point toward TCA enzyme activity reductions, resulting in reduced cerebral glucose metabolism, which translates to clinically relevant cognitive impairments.

While age is the primary risk factor for AD, genetic factors also strongly contribute to disease risk (Bertram et al., 2007; Bertram et al., 2009). The strongest genetic risk factor for the more common late-onset form of AD is *APOE* [98,99]. In humans, there are three major isoforms of apoE: E2, E3, and E4 [100]. Apolipoprotein E (apoE), which is associated with circulating lipoproteins, plays several important metabolic roles [100]. In the periphery, apoE is primarily produced by the liver, while in the brain, apoE is primarily produced by astrocytes, and is responsible for neuronal maintenance and repair in addition to a number of other physiological and pathological roles [101,102,103]. E3 is the major isoform expressed in humans (~60% of the population), and the effects of E2 and E4 are typically compared to those of E3 to determine the relative risk [98,104]. Importantly, the E4 isoform confers between a 2- (heterozygous) to 15-fold (homozygous) increase in the risk of developing AD [98], and studies suggest the E4 allele may account for up to 50% of AD in the US [99]. 

Interestingly, a consistent pattern of brain glucose hypometabolism, similar to that seen in AD, has been noted in individuals with E4 [84,105]. Even non-demented, cognitively normal E4 carriers demonstrate this pattern of glucose hypometabolism [106]. Importantly, these metabolic deficits are present decades in advance of AD onset in E4+ and other at-risk individuals thereby lending support to this being an inherent biological feature of E4, rather than simply a byproduct of dementia [107,108]. The metabolic impairments associated with E4 extend beyond reductions in CMRglc to also include mitochondrial deficits described in several in vivo and in vitro models. For example, the expression of the subunits of several mitochondrial respiratory complexes is decreased in neurons expressing E4 compared to those expressing E3 in a mouse model of neuron-specific apoE expression [109]. Mitochondrial protein expression was also shown to be modified by *APOE* genotype in both the absence and presence of an ischemic injury [110]. Consistent with a role of upregulated mitochondria-associated endoplasmic reticulum membrane (MAM) function in AD, Tambini et al. recently showed that E4-containing astrocyte conditioned media increased MAM activity in vitro [111]. Additionally, a series of studies in mice show that apoE4 is cleaved by a protease in neurons to generate a toxic apoE4(1-272) fragment with a mitochondrial targeting sequence [112]. This fragment of apoE4 has been shown to bind to mitochondrial complexes and affect their activities [113]. Finally, an exciting new study by Zhao et al. shows that E4 inhibits insulin-induced mitochondrial respiration in primary neurons [114].

Two studies have reported an interaction between *APOE* genotype and mitochondrial DNA haplogroups in determining AD risk [115,116], and E4-associated mitochondrial deficits have also been confirmed in human autopsy tissue. Importantly, these abnormalities appear to begin early in life—at a point that precedes amyloid deposition. For example, post-mortem brains from young adult *APOE* ε4 carriers show reduced COX [117,118]. Interestingly, these findings extend to the periphery, as platelet mitochondria COX activity in AD subjects with E4 alleles are lower than in non-carrier AD subjects [119]. 

In addition to Alzheimer’s disease, mitochondrial dysfunction has been reported in other neurodegenerative diseases such as Huntington’s disease, Parkinson’s disease, and amyotrophic lateral sclerosis. Mitochondrial metabolism in these diseases has been extensively reviewed previously [120,121,122,123,124]; therefore, we will be focusing on other neurological disorders such as traumatic brain injury and epilepsy for the remainder of the review.

## 3. Mitochondrial Disruption Following TBI 

Traumatic brain injury (TBI) is a major cause of death and disability estimated to affect approximately 1.7 million people annually in the US [125]. The cause of injury consists of a primary insult due to acceleration and deceleration forces on neuronal structures. Following the primary injury phase is the secondary injury cascade. It is believed that it is the secondary injury, involving a complex cascade of biochemical, metabolic, and molecular changes, that leads to profound malfunctioning in cerebral cells and permanent long-term sequelae of TBI. Importantly, metabolic changes are heavily involved in this process [126,127,128]. It has been proposed that it’s the neuronal hyperexcitability, intracellular calcium concentration elevations, and mitochondrial enzymatic alterations in the secondary cascade that ultimately lead to oxidative stress and cellular death [129,130,131].

The secondary injury cascade after TBI is initiated by disruptions in the membrane potential that lead to spontaneous firing of the neuronal cells [132]. The aberrant action potentially causes the release of numerous neurotransmitters, primarily the excitatory amino acid glutamate [133,134]. Increases in extracellular glutamate concentrations following TBI are accompanied by a rise in lactate and a decline in extracellular glucose [135,136,137], suggestive of a shift from oxidative metabolism to non-oxidative methods such as glycolysis. Glutamate is an amino acid that is closely intertwined with mitochondrial energy production [138]. Its biosynthesis takes place in the mitochondria, from either αKGDH by GDH or from glutamine by GLS. Furthermore, the cytoplasmic enzyme GOT promotes the reversible conversion of glutamate to αKGDH, a key step during nucleotide biosynthesis. The aberrant release of glutamate could potentially alter all of the linked pathways described above. In addition, glutamate is one of the precursors for the production of gamma-aminobutyric acid (GABA), the major inhibitory neurotransmitter [139,140]; hence, an ectopic release of glutamate could result in the lack of GABA production, and consequently, a lack of inhibitory responses in the brain. The interplay between glutamate, the TCA cycle, and mitochondrial metabolism is only beginning to emerge. Due to the complexity of the metabolic networks affected by glutamate, further metabolomic approaches are needed to improve our understanding at a system level. 

The increase in excitatory neurotransmission from increased glutamate release leads to cellular depolarization and calcium influx into neurons via AMPA, NMDA channel, and voltage-gated calcium channels (VGCC) [141,142,143], further causing energy depletion and excitotoxic-mediated cell death [144]. As the intracellular calcium concentration increases, the mitochondria attempt to buffer intracellular calcium by sequestering it into the matrix through mitochondrial permeability transition pores [143,145]. Increased mitochondrial calcium levels result in mitochondrial dysfunction in a dose-dependent manner [146,147,148]. Furthermore, studies have shown that increased calcium levels have an inhibitory effect over key mitochondrial dehydrogenase enzymes such as PDH, NAD-dependent isocitrate dehydrogenase, and αKGDH in in vitro models [149,150]. Several studies have confirmed these findings after TBI. PDH activity has been found to be significantly reduced in blood levels of TBI patients [151]. Similarly, in a rodent model, αKGDH activity was decreased after TBI, while application of a αKGDH coenzyme precursor, thiamine, rescued the activity of αKGDH and restored mitochondrial respiration [152]. Further studies are needed to confirm the effect of calcium elevations on other mitochondrial enzymes in TBI. 

In addition to the aforementioned increase in excitatory signaling and intracellular calcium, several other factors may contribute to the energy crisis defined after TBI. HIF1α, a transcription factor responsive to acute hypoxia, has been found to be elevated in the brains of mice after TBI models [153,154]. Multiple studies have documented a significant role of HIF1α in elevating aquaporin (AQP)-4 and -9 further exacerbating brain edema post-TBI [153,154,155]. Pharmacological inhibition of HIF1α using 2-methoxyestradiol (2ME2) reduced brain edema, AQP-4 and -9 expression [153]. HIF1α activation also reduces glucose and glutamine flux through mitochondria in other experimental models [156,157,158]; however, it has not yet been investigated whether similar metabolic changes are also occurring post-TBI. 

It has been long shown that acute mitochondrial disruption occurs following experimental TBI [142,159]. The secondary injury cascade after TBI causes detrimental cell damage and is marked by alterations in excitatory amino acids such as glutamate [160,161] increases in acute hypoxia [162], and the disruption of calcium homeostasis [141]; all of which alter mitochondrial metabolism. The vital roles for mitochondria in cellular function and survival have resulted in increased efforts to identify the molecular events associated with mitochondrial impairment in TBI.

## 4. Role of Mitochondria in Epilepsy

Epilepsy is a neurological disorder that affects approximately 1.2% of the US population [163]. It consists of abnormal, excessive, hypersynchronous discharges from a population of neurons that disrupt normal brain function. The causes of epilepsy are multifactorial, ranging from trauma to the brain, tumor growth, infection, malformations, or genetic abnormalities [164]. Despite the large variability in causes, mitochondrial dysfunction is frequently involved. Mitochondrial gene mutations frequently lead to seizures [165,166], while nuclear genes that alter mitochondrial function can also result in epilepsy [167]. Moreover, seizure activity triggers the release of ROS, calcium influxes, neurotransmitter imbalances that cause further alterations in mitochondrial metabolism, and neuronal death [168,169,170]. Therefore, mitochondrial dysfunction can be both a cause and a consequence of epileptic disease [171]. 

Research in the last few decades has highlighted numerous nuclear genes that alter mitochondrial function, over 100 of which have been linked to epilepsy [167]. Mutations in the nuclear gene POLG, which encodes for the catalytic subunit of mitochondrial DNA, polymerase gamma, has been the one of the most studied [172,173,174,175,176,177]. Mutations have resulted in numerous phenotypes ranging from generalized seizures to myopathy, hepatopathy, sensory-ataxias, and opthalmoparesis [175,178]. Iron–sulfur clusters are important for the assembly of the electron transfer complexes [179]. Disruption of multiple genes in this pathway, such as LIAS, BOLA3, and NFU1, are associated with mitochondrial-disease-induced epilepsy [180,181,182]. Furthermore, mutations in mitochondrial transporter genes SLC25A12 and SLC25A22 are another cause of epilepsy [183,184]. These are members of the solute carrier family 25 and are both used for intracellular glutamate transport between the mitochondria and the cytoplasm. Aberrant function of these transporters is thought to be responsible for excessive glutamate release leading to hyperexcitability [185,186,187]. Additional nuclear genes that alter mitochondrial function and have been shown to be associated with epilepsies have been listed in Table 1. 

The mitochondrial genome is composed of circular, double-stranded DNA containing 37 genes which are all essential for the normal function of the mitochondrion. While mtDNA represents only a small fraction of total human DNA, mutations in over half of the 37 mtDNA genes have been associated with epilepsy [167]. The most frequently occurring mutations are in mitochondrial tRNA genes that lead to the epileptic syndromes such as mitochondrial epilepsy, lactic acidosis, stroke-like episodes (MELAS), and myoclonic epilepsy with ragged red fibers (MERRF) [252,253]. Less commonly seen mutations involving oxidative phosphorylation complexes also can induce various epileptic syndromes [167,254]. For example, mutations in mitochondrial genes that result in encoding proteins forming complexes I, III, IV, and V have been noted to cause Leigh syndrome, a mitochondrial disorder resulting in a severe, progressive, necrotizing encephalopathy [196]. While the syndrome is characterized by motor dysfunction and intellectual regression in the first few years of life, epilepsy is observed in 21–39% of patients [255,256]. 

Mitochondrial dysfunction and seizures have been found to be closely linked not only in genetic causes of epilepsy, but are also becoming increasingly recognized in acquired epilepsy [167]. These non-genetic causes of epilepsy include physical head trauma, infectious diseases, brain tumors, or drug intoxications [257]. Irrespective of the trigger, seizure activity significantly decreases ATP levels in neurons, suggestive of energy depletion [258]. Studies have identified that synaptic transmission is the largest consumer of neuronal ATP [259,260]; therefore, with the excessive neuronal firing during seizures, it is not surprising that energy depletion occurs leading to metabolic dysfunction even in acquired epilepsies. Other characteristics of homeostatic changes occurring after seizures are the accumulation of intracellular calcium and ROS [261,262,263]. Excess ROS and Ca^2+^ are potent triggers causing the opening of the mitochondrial permeability transition pore, leading to irreversible mitochondrial swelling and cytochrome c release, subsequently triggering mitochondrial damage and cellular death [264,265]. Due to a neuron’s lack of glycolytic capacities, the metabolic strain post-seizure activity increases the demand for oxidative phosphorylation and is a possible trigger for the excess ROS and Ca^2+^ fluctuations. Furthermore, oxidative damage can have profound effects on neurotransmitter uptake and release, altering neuronal excitability [266] and further increasing the likelihood of seizure activity and exacerbating neuronal energy depletion [267]. 

Regardless of which came first, epilepsy and mitochondrial dysfunction interact in a vicious cycle to deplete a neurons’ energy stores, leading to extensive brain damage. Although a detailed connection between mitochondrial dysfunction, energy metabolism, and epilepsy remains to be elucidated, mitochondria are clearly an integral part of disease manifestation.

## 5. Concluding Remarks

AD, TBI, and epilepsy are just a few of the neurological diseases with mitochondrial involvement. Metabolic dysfunction has also been documented in numerous other neurological diseases such as stroke [268,269], glioblastomas [270], multiple sclerosis [271,272], various neurodegenerative diseases [120,121,122,123,124], and even psychiatric disorders [273,274,275]. In this article, we have highlighted the importance of mitochondria metabolism in cellular pathophysiology in a few of the major neurological disorders and hope to have shed light on areas of exploration to further unravel the complexities of mitochondrial involvement in disease states. 

Mitochondrial metabolism supplies the energy demand of a cell, coordinates the balance between catabolism and anabolism, and maintains redox homeostasis. However, knowledge gaps still remain to elucidate detailed mechanisms that lead to the manifestation of disease. While high-resolution techniques, such as chromatograph-based mass spectrometry metabolomics methods, have provided an efficient way to visualize mitochondrial metabolism in different types of cancers, these methods have not been adopted in the field of neurological research. Future research should incorporate traditional metabolomics and stable tracer technology to map mitochondrial metabolic flux with high-resolution, to yield vital information for the understanding of disease pathophysiology, and to assist in the design of future therapeutic agents to treat neurological disorders. 

## Figures and Tables

**Table 1:** Nuclear genes involved in mitochondrial function associated with epilepsy.

	Gene	Source
Complex I Subunits Assembly factors	*NDUFS1, NDUFS2, NDUFS3, NDUFS4, NDUFS6, NDUFS7, NDUFS8, NDUFV1, NDUFA1, NDUFA11*	[188,189,190,191,192,193,194,195,196]
Complex I Assembly Factors	*NDUFAF1, NDUFAF2, NDUFAF3, NDUFAF4, NDUFAF5, NDUFAF6, AIFM1, FOXRED1, NUBPL*	[197,198,199,200,201,202,203]
Complex II Subunits	*SDHA*	[204]
Complex III Subunits and Assembly Factors	*UQCC2, UQCRC2, BCS1L*	[205,206,207]
Complex IV Subunits and Assembly Factors	*NDUFA4, ETHE1, APOPT1, SURF1, SCO1, SCO2, COX10, COX15, FASTKD2, PET100*	[208,209,210,211,212,213,214,215,216]
Complex V Subunits and Assembly Factors	*ATP5A1, ATPAF2, TMEM70*	[210,217,218]
Solute Carriers	*SLC25A12, SLC25A22*	[183,184]
Coenzyme Q10 Biosynthesis	*ADCK3, COQ2, COQ4, COQ6, COQ9, PDSS2*	[219,220,221,222,223,224]
ETC Cofactor Biosynthesis	*LIAS, BOLA3, NFU1, NFS1, TPK, HCCS*	[181,182,225,226,227,228]
tRNA Aminoacylation	*AARS2, CARS2, DARS2, EARS2, FARS2, NARS2, RARS2, VARS2*	[229,230,231,232,233,234,235,236]
Posttranslational Regulators	*GFM1, TSFM, PNPT1, RMND1, TRIT1, TRNT1*	[237,238,239,240,241,242]
Mitochondrial DNA Replication	*C10orf2, POLG*	[197,243]
Nucleotide Pool	*DGUOK, TYMP, TK, RRMB*	[244,245,246]
TCA Cycle	*SUCLA2, SULG1, SDHAF1*	[247,248,249]
Other Mitochondrial DNA Depletion Syndromes	*FBXL4, MPV17*	[250,251]
